# Supplementary material for: Identification of photoperiod-induced specific miRNAs in the adrenal glands of Sunite sheep (Ovis aries)
Source: Front Vet Sci. 2022 Jul 22;9:888207. doi: 10.3389/fvets.2022.888207 (PMC9354845; doi:10.3389/fvets.2022.888207)
Supplement: Supplementary file 1 [file Data_Sheet_1.ZIP › Supplementary file/miRDeep2 file.zip!/miRDeep2 file/Know_Structure/LP42A2/oar-mir-26b.pdf]

miRBase precursor	: oar-mir-26b
Total read count	: 124710
oar-miR-26b read count	: 124573
remaining reads	: 137

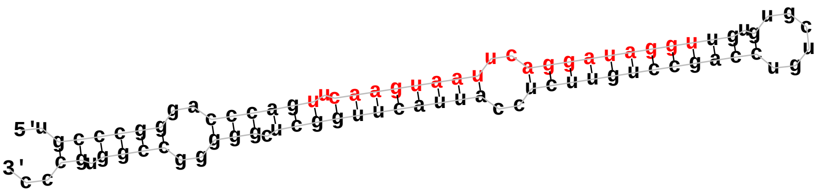

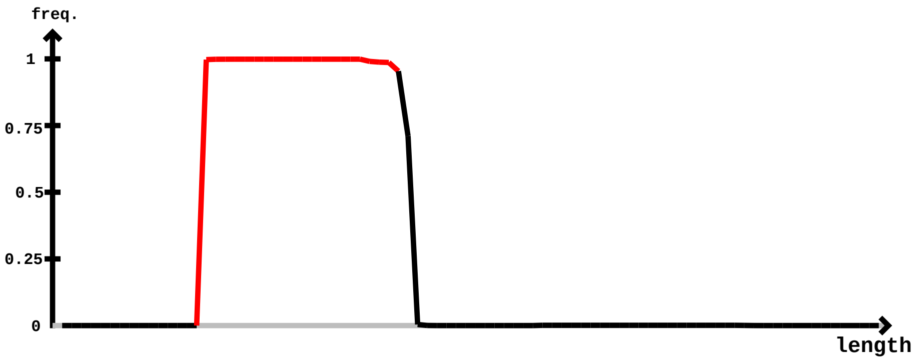

oar-miR-26b

```
5'- ugcccgggacccaguucaaguaauucaggauagguugugugcuguccagccuguucuccauuacuuggcucgggggccggugccc  -3'    exp
    .((((((..((((((.((((((((..(((((((((((.(......))))))))))))..))))))))))).))).)))).))..    reads     mm        sample
    .............guucaaguaauucaggauagguu.................................................    1         0         seq
    ..............uucaaguGauucaggau......................................................    1         1         seq
    ..............uucCaguaauucaggau......................................................    56        1         seq
    ..............uucaaguaauucaggaG......................................................    22        1         seq
    ..............uucaaguaauucaggUu......................................................    3         1         seq
    ..............uucaaguaauuUaggau......................................................    1         1         seq
    ..............uucaUguaauucaggau......................................................    1         1         seq
    ..............uuUaaguaauucaggau......................................................    1         1         seq
    ..............uucaaguaauucagCau......................................................    1         1         seq
    ..............uucaaguaauucaggaA......................................................    24        1         seq
    ..............uucaaguaauucaggCu......................................................    2         1         seq
    ..............uucaGguaauucaggau......................................................    2         1         seq
    ..............uucaaguaaCucaggau......................................................    1         1         seq
    ..............uucaaguaauucaggau......................................................    788       0         seq
    ..............Aucaaguaauucaggau......................................................    5         1         seq
    ..............uucaaguaauucaggaC......................................................    123       1         seq
    ..............uCcaaguaauucaggau......................................................    3         1         seq
    ..............uucaaguaauucaggaua.....................................................    327       0         seq
    ..............uucaaguaauucaggaCa.....................................................    1         1         seq
    ..............uucCaguaauucaggaua.....................................................    15        1         seq
    ..............uucaaguaauucaggaAa.....................................................    4         1         seq
    ..............uucaaguaauucaggauag....................................................    166       0         seq
    ..............uucaaguaauucaggauagg...................................................    4042      0         seq
    ..............uucaaguaauucaggauaggu..................................................    30298     0         seq
    ..............uucaaguaauucaggauagguu.................................................    87977     0         seq
    ..............uucaaguaauucaggauagguug................................................    464       0         seq
    ..............uucaaguaauucaggauagguugu...............................................    43        0         seq
    ...............ucaaguaauucaggauC.....................................................    1         1         seq
    ...............ucaaguaauucaggauag....................................................    1         0         seq
    ...............ucaaguaauucaggauagg...................................................    2         0         seq
    ...............ucaaguaauucaggauaggu..................................................    38        0         seq
    ...............ucaaguaauucaggauagguu.................................................    118       0         seq
    ...............ucaaguaauucaggauagguug................................................    8         0         seq
    ................caaguaauucaggauagg...................................................    1         0         seq
    ................caaguaauucaggauaggu..................................................    6         0         seq
```

oar-miR-26b

ugcccgggacccaguucaaguaauucaggauagguugugugcuguccagccuguucuccauuacuuggcucgggggccggugccc			
................caaguaauucaggauagguu...	15	0	seq
.................aaguaauucaggauaggu..	1	0	seq
.................aaguaauucaggauagguu...	7	0	seq
..................aguaauucaggauaggu..	1	0	seq
..................aguaauucaggauagguu...	2	0	seq
....................................gugugcuguccagccuguucuccauuacuuggcuc..............	1	0	seq
....ccuguucuccauuacuuggc................	3	0	seq
....ccuguucuccauuacuuggcu...............	58	0	seq
....ccuguucuccauuacuuggcuc..............	67	0	seq
....ccuguucuccauuacuuggcucg.............	7	0	seq
....cuguucuccauuacuuggcu...............	1	0	seq
